# Estimating Potential Eligibility for Disease Modifying Therapies for Alzheimer’s Disease Using Population‐Based Data in Alberta, Canada

**DOI:** 10.1002/alz.085900

**Published:** 2025-01-09

**Authors:** Mohammad Z I Chowdhury, Gavin Thomas, Eric E. Smith, Susan E Bronskill, Julia G Kirkham, Zahinoor Ismail, Zahra Goodarzi, Pamela Roach, Vivian Ewa, Isabelle Vedel, Andrea Gruneir, Dallas Seitz

**Affiliations:** ^1^ University of Calgary, Calgary, AB Canada; ^2^ Department of Clinical Neurosciences and Hotchkiss Brain Institute, University of Calgary, Calgary, AB Canada; ^3^ Hotchkiss Brain Institute, University of Calgary, Calgary, AB Canada; ^4^ ICES, Toronto, ON Canada; ^5^ University of Toronto, Toronto, ON Canada; ^6^ McGill University, Montreal, QC Canada; ^7^ University of Alberta, Edmonton, AB Canada

## Abstract

**Background:**

Disease modifying therapies (DMTs) for Alzheimer’s disease (AD) have been approved in some countries although these treatments will require substantial health resources for their implementation. Initial capacity planning to identify the resources required to support DMTs begins with estimating the number of people with dementia who may be eligible for DMTs. We estimated the potential number of individuals with dementia who are eligible for DMTs using population‐based data in Alberta, Canada.

**Method:**

We used provincial administrative health databases in Alberta, Canada (population ∼4.5 million) to identify all people newly identified with dementia between April 1, 2013 and March 31, 2022 using a validated dementia case ascertainment algorithm. The characteristics of this population were then described at the time of diagnosis including demographics, conditions potentially contributing to the diagnosis of dementia (e.g. stroke, parkinsonism), and medical contraindications to treatment (e.g. cancer, seizures). The characteristics of individuals with dementia were then compared to the inclusion criteria used in AD DMT trials to estimate the proportion of individuals with dementia who may be eligible for DMTs. We then compared the characteristics of individuals who were determined to be eligible for DMTs compared to those who were not eligible.

**Result:**

A total of 77,479 individuals were identified with incident dementia during the study period with a mean age of 80.6 years and 58% were female. In this group, 15,044/77,479 (19%) had conditions other than AD as a potential cause of their dementia. Medical contraindications to DMTs were present in 24,580/62,435 (39%) of all individuals who had AD as their probable cause of dementia. Overall, 37,855/77,479 (49%) of the entire dementia population could potentially go on to receive biomarker testing to determine DMT eligibility. Individuals who were younger and women were more likely to be potentially eligible for DMTs at the time of dementia diagnosis.

**Conclusion:**

Approximately half of all people diagnosed with dementia in Alberta could be eligible for DMTs based on their health history. Additional information on cognitive test scores, neuroimaging, and biomarker results are required to further refine estimates of the number of individuals potentially eligible to receive DMTs in Canada.

